# Central nervous system involvement of acute promyelocytic leukemia, three case reports

**DOI:** 10.1002/ccr3.919

**Published:** 2017-03-29

**Authors:** Aya Furuya, Masahiro Kawahara, Mina Kumode, Yasuyuki Ohira, Asako Usui, Shiho Nagai, Sakura Hosoba, Hitoshi Minamiguchi, Katsuyuki Kito, Akira Andoh

**Affiliations:** ^1^ Division of Gastroenterology and HematologyDepartment of MedicineShiga University of Medical ScienceOtsuJapan

**Keywords:** Acute promyelocytic leukemia, arsenic trioxide, central nervous system involvement, triple intrathecal treatment

## Abstract

Central nervous system (CNS) involvement of acute promyelocytic leukemia (APL) causes poor prognosis. Our three cases show that CNS can be involved at the first hematological recurrence, but predicting this is difficult. Triple intrathecal treatment and craniospinal irradiation were effective, while arsenic oxide failed to prevent and improve CNS involvement.

## Introduction

Acute promyelocytic leukemia (APL) is a highly curable disease in the era of all‐trans retinoic acid (ATRA) but still approximately 10% to 20% of cases relapse 1, 2, 3. When there is central nervous system (CNS) involvement, eradication of abnormal promyelocytes from the patients is difficult. As a result, median overall survival (OS) is shorter in patients with CNS involvement than that in patients with hematological recurrences only 4. Risk classifications such as Sanz score 5 are frequently used; however, their predictive usefulness for CNS involvement is unclear. Although several novel drugs such as arsenic trioxide (ATO) and tamibarotene (Am80) have been developed and used clinically 6, 7, their efficacy against abnormal promyelocytes that infiltrate into CNS remains unclear. In this case series, we present three cases of APL with CNS involvement experienced in our institution. Informed consent was obtained from three patients whose cases are discussed. Through describing the status at initial diagnosis and the clinical courses, in particular, treatment histories around the time of diagnosis of CNS involvement, we discuss whether clinical or laboratory findings could predict a higher chance of CNS involvement and what treatments are effective to eradicate abnormal promyelocytes from CNS.

## Case Report

### Case 1

**Figure 1:**
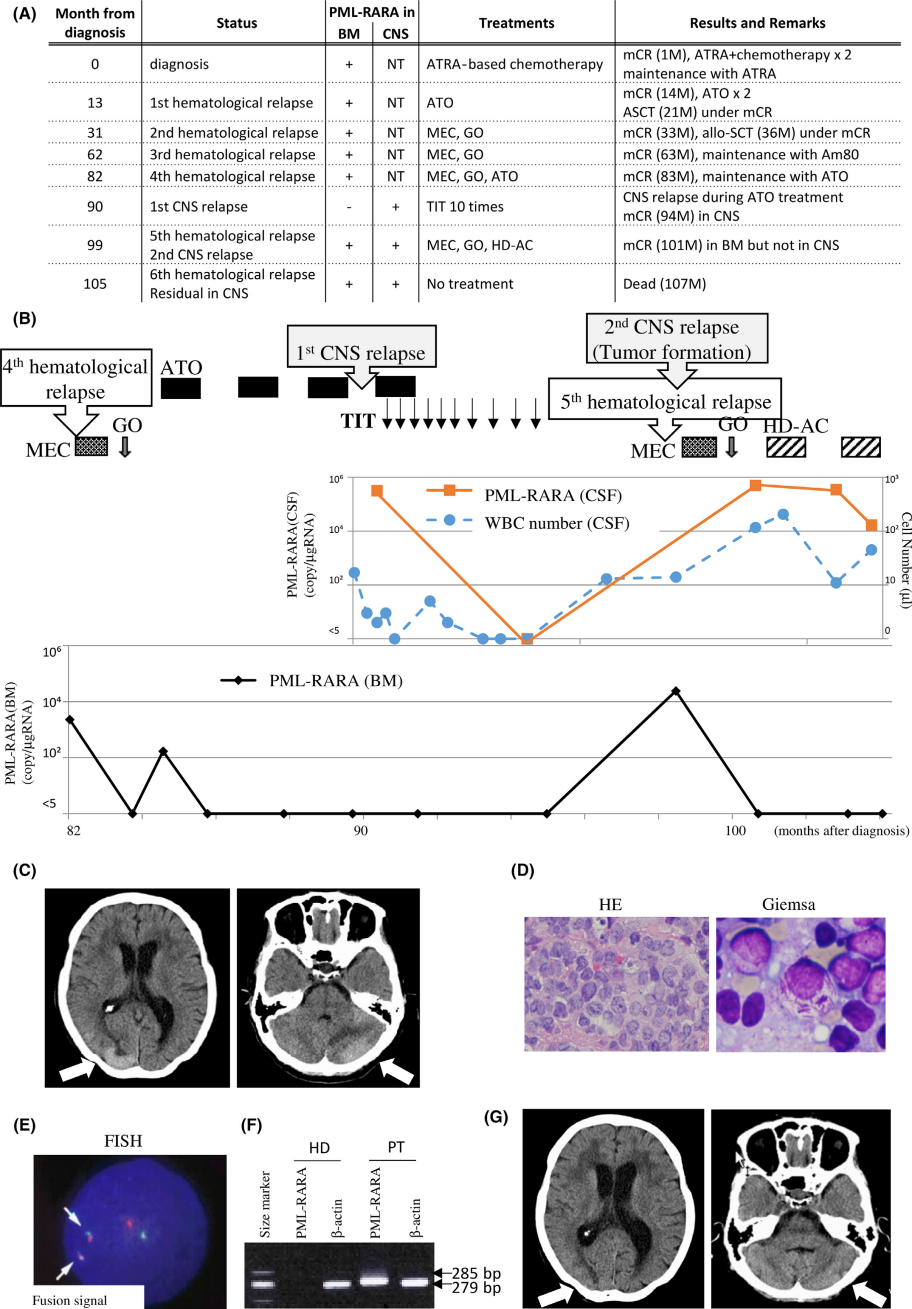
Case 1 presentation. (A) Entire clinical course. CNS, central nervous system; BM, bone marrow; TIT, triple intrathecal therapy; mCR, molecular complete remission; ATRA, all‐trans retinoic acid; ASCT, autologous stem cell transplantation; allo‐SCT, allogeneic stem cell transplantation; NT, not tested. (B) Clinical course of case 1 after fourth relapse that occur at 98 months or 62 months after initial diagnosis or allo‐SCT, respectively. The provided treatments are shown in the upper. Graphs show PML‐RARA copy number in the BM or CSF, and WBC number in the CSF as indicated. (C) Head CT analysis when bleariness occurred. Tumor formation was observed in the left hemisphere of cerebellum and the right occipital lobe cortex. Both lesions are indicated by arrows. (D) Morphologic analyses of the biopsy sample from the CNS tumor by hematoxylin and eosin staining (HE, left, 400 × ) and Giemsa staining (right, 1000×). (E) Fluorescence in situ hybridization (FISH) analysis of the WBC cells from the CSF. 98% cells were fusion signal positive in the CNS tumor biopsy sample. The PML probe and the RARA probe are labeled with red and green, respectively. Arrows indicate fusion signals. (F) Reverse transcription polymerase chain reaction (RT‐PCR) of PML‐RARA. The 285‐bp band indicates the bcr3 type of the PML‐RARA fusion gene. The 279‐bp band indicates an internal control. HD and PT mean a healthy donor and the patient, respectively. (G) Head CT analysis before the treatment with HD‐AC. Tumors indicated by arrows were reduced by MEC and GO.

The patient was 57‐year‐old female at the initial diagnosis. Peripheral white blood cell (WBC) count was 0.8 × 10^9^/L, platelet count was 66 × 10^9^/L, and her bone marrow was filled with abnormal promyelocytes up to 85.6%. Chromosomal analysis revealed 46 XX,t(15,17)(q22;q21) without additional chromosomal abnormalities. The PML‐RARA fusion gene transcript was also detected, and its isoform was bcr3. Based on these findings, she was diagnosed with APL classified in the low‐risk group by Sanz score 5. The entire clinical course is summarized in Figure 1A. In, brief, ATRA plus chemotherapy (ATRA 45 mg/m^2^, idarubicin (IDA) 12 mg/m^2^ for 2 days and cytarabine 100 mg/m^2^ for 5 days) was administrated and successfully induced molecular complete remission (mCR) without CNS hemorrhage. However, the patient relapsed at 13 months after the initial diagnosis during an ATRA‐based maintenance therapy following three courses of consolidation therapies. ATO was intravenously administrated in the dose of 0.15 mg/kg/day every day until second mCR was achieved. Although autologous stem cell transplantation (ASCT) was performed after two additional courses of ATO (0.15 mg/kg/day in 5 days a week for 5 weeks), the patient relapsed 10 months later. The MEC regimen (MEC; mitoxantrone 6 mg/m^2^ on days 1–4, etoposide 80 mg/m^2^ on days 1–5, and acytarabine 100 mg/m^2^ on days 1–5) and gemtuzumab ozogamicin (GO 3 mg/m^2^) induced third mCR. In addition, allogeneic stem cell transplantation (allo‐SCT) from a HLA‐matched unrelated donor was performed. However, 26 months later, the patient relapsed for the third time. The MEC regimen and GO treatment were re‐administrated and induced fourth mCR. Prevention of relapse was maintained by subsequent administration of tamibarotene (Am80), but was only successful in doing this for 19 months, after which she had fourth time hematological relapse. Re‐administration of ATO following MEC and GO successfully induced and maintained mCR for 7 months. However, during this period, cerebrospinal fluid (CSF) test that was performed despite no neurological symptoms revealed slight increase in the WBC number and clear detection of the PML‐RARA fusion gene transcript by quantitative PCR in CSF cells, indicating that abnormal promyelocytes had infiltrated into CNS (Fig. 1B). Triple intrathecal therapy (TIT; methotrexate 15 mg, cytarabine 40 mg and prednisolone 10 mg) proved highly effective in reducing the quantity of abnormal promyelocytes in CSF and finally resulted in mCR in CNS. Four months following the last TIT, the patient had fifth hematological relapse. Although no new chromosomal abnormalities were detected, the patient simultaneously indicated that she was experiencing visual disturbances. Computed tomography (CT) demonstrated tumor formation in the left hemisphere of cerebellum and the right occipital lobe cortex (Fig. 1C). To exclude a possibility of cranial nerve tumors, biopsy samples were analyzed morphologically and genetically. Hematoxylin and eosin (HE) staining revealed monotonous expansion of abnormal cells, and Giemsa staining clearly exhibited the existence of faggot cells (Fig. 1D). Fluorescence in situ hybridization (FISH) demonstrated the proliferation of cells with fusion signals of PML and RARA up to 98% in CSF (Fig. 1E). PCR demonstrated that the PML‐RARA transcript isoform is bcr3 (Fig. 1F), indicating that the brain tumor was a result of the abnormal promyelocytes. Retreatment with MEC and GO improved her visual disorder and reduced tumor volume (Fig. 1G), but high copy number of PML‐RARA was still detected in CSF. Two courses of high‐dose cytarabine therapy (HD‐AC; 1500 mg/m^2^ for 5 days) were additionally administrated but were no longer able to eliminate abnormal promyelocytes from CNS. Finally, during cessation of treatments owing to poor systemic condition caused by heavily repeated therapies, APL clones rapidly expanded in CNS and bone marrow (BM) again. As all therapies had been discontinued, the patient died 27 months after her first CNS relapse.

### Case 2

**Figure 2:**
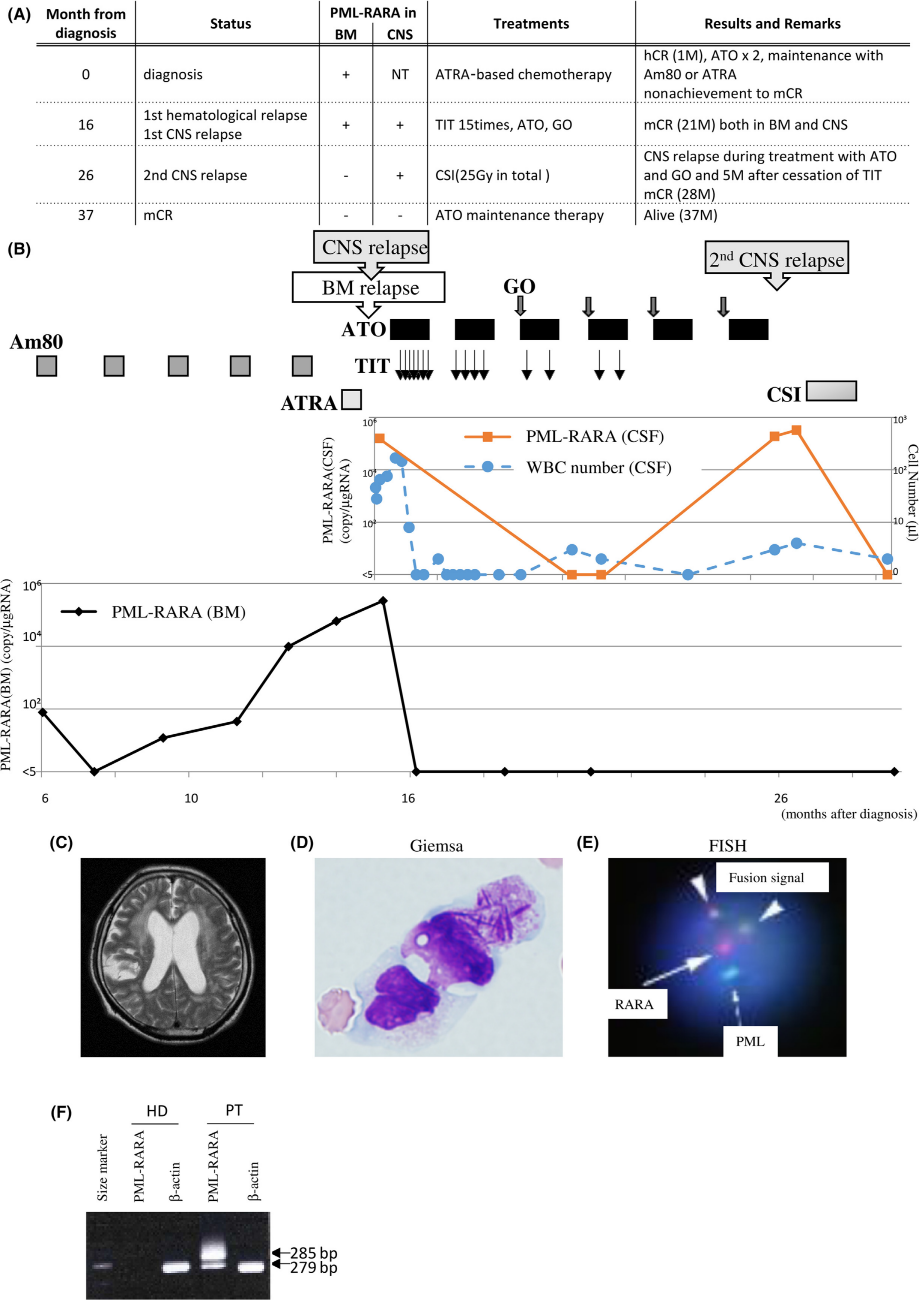
Case 2 presentation. (A) Entire clinical course. Abbreviations are as shown in Figure 1A. (B) Clinical course of case 2 after starting Am80 treatment. The provided treatments are shown in the upper panel. Graphs show PML‐RARA copy number in the BM or CSF, and WBC number in the CSF as indicated. (C) Head CT analysis when neurological symptoms became clinically evident. Hydrocephalus was observed. (D) Morphologic analyses of the WBC cells from the CSF by Giemsa staining (1000 × ). (E) FISH analysis of the WBC cells from the CSF. 91.9% cells were fusion signal positive in the CNS tumor biopsy sample. The PML probe and the RARA probe are labeled with green (arrow) and red (dotted arrow), respectively. Triangles indicate fusion signals. (F) RT‐PCR of PML‐RARA. The 285‐bp band indicates the bcr3 isoform of the PML‐RARA fusion gene. The 279‐bp band indicates an internal control.

The patient was 68‐year‐old male at the initial diagnosis. Peripheral WBC count was 3.4 × 10^9^/L, platelet count was 10 × 10^9^/L, and his bone marrow was occupied with abnormal promyelocytes up to 90.4%. He had no findings of cerebral hemorrhage despite of severe bleeding symptoms such as purpura and oral bleeding due to disseminated intravascular coagulation (DIC). Chromosomal analysis revealed 46 XX, t(15;17)(q22;q21) without additional chromosomal abnormalities. The PML‐RARA fusion gene transcript was also detected, and its isoform was bcr3. Based on these findings, he was diagnosed with APL classified in the intermediate risk group by Sanz score 5. The entire clinical course is summarized in Figure 2A. In brief, ATRA plus IDA (ATRA 5 mg/m^2^ orally daily and IDA 8 mg/m² for 3 days) was administrated as induction therapy and successfully induced hematological CR. Subsequently, two consolidation courses of ATO (0.15 mg/kg/day in 5 days a week for 5 weeks) and maintenance with Am80 (6 mg/m² orally 14 consecutive days every 3 months) were performed to avoid the toxicity associated with the chemotherapy. The PML‐RARA fusion gene transcript once disappeared from the BM after one course of Am80 but increased again in the BM during repeated courses of Am80. The patient experienced a hematological relapse without additional chromosomal abnormalities 16 months after the initial diagnosis (Fig. 2B). Simultaneously, he presented with neurological symptoms such as cognitive functional disorder and walk disorder. Head CT revealed hydrocephalus (Fig. 2C), and CSF test clearly showed the existence of faggot cells in CSF (Fig. 2D). FISH demonstrated cells with fusion signals between PML and RARA up to 91.9% of CSF cells (Fig. 2E). PCR demonstrated that the PML‐RARA transcript isoform is bcr3 (Fig. 2F), indicating that abnormal promyelocytes infiltrated into the CNS. Thus, in addition to a re‐induction therapy with ATO, TIT (methotrexate 15 mg, cytarabine 40 mg and prednisolone 10 mg) was administrated once a week until abnormal promyelocytes disappeared from the CSF. The PML‐RARA transcript disappeared from the BM after one course of ATO, and from the CSF after three courses of ATO plus 13 times of TIT. GO (4 mg/m^2^ on day 1 of the ATO course) and ATO were alternately administrated as a consolidation therapy after second course of ATO. TIT was ceased from fifth course of ATO because of the patient's request. However, the PML‐RARA transcript level increased in the CSF after sixth course of ATO, despite maintenance of mCR in the BM. Finally, the patient received craniospinal irradiation (CSI; 25 Gy in total) that was highly effective in reducing his CNS residual disease. Currently the patient retains mCR in both the BM and CNS without any neurological complications for 9 months since completing CSI.

### Case 3

**Figure 3:**
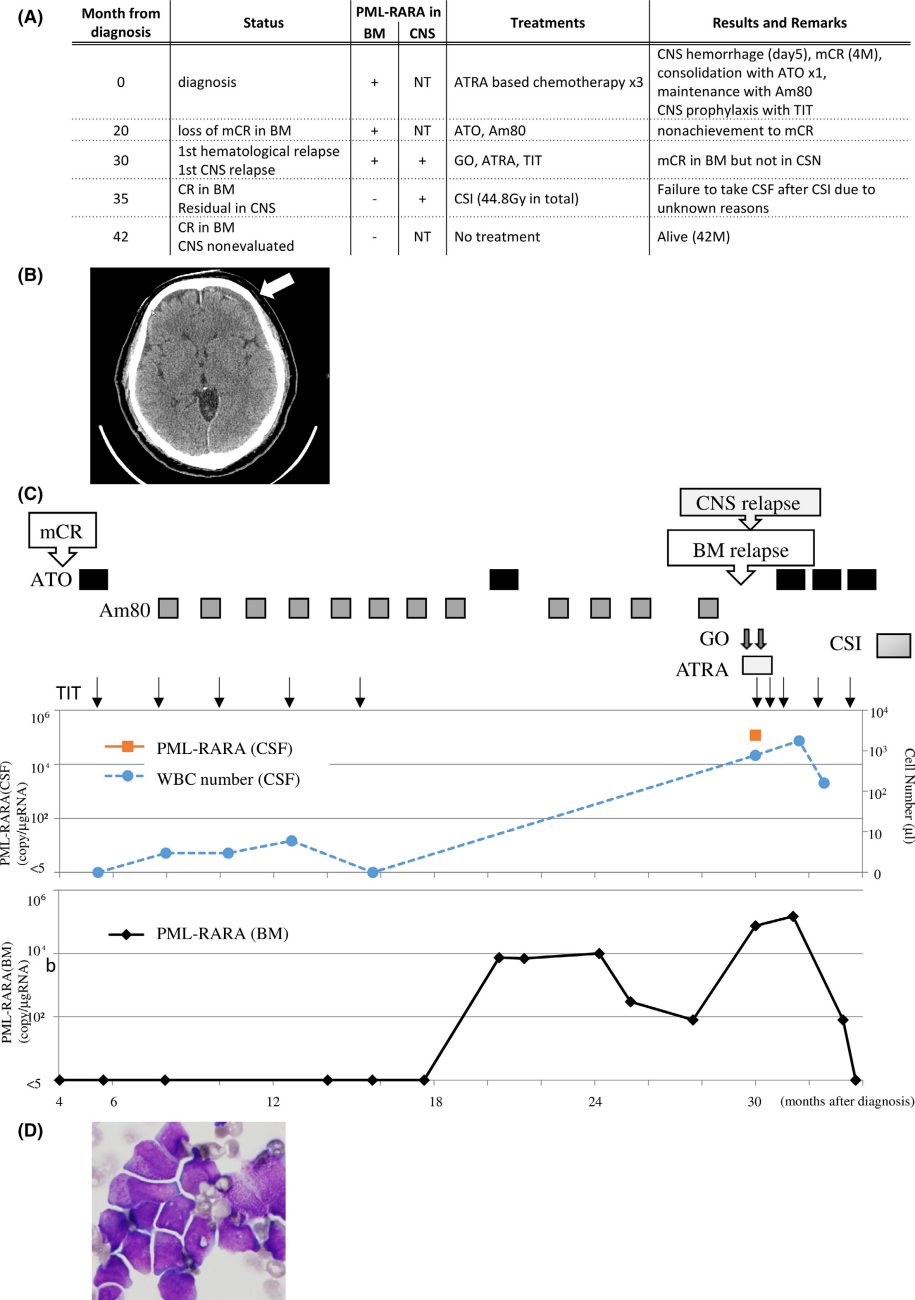
Case 3 presentation. (A) Entire clinical course. Abbreviations are as shown in Figure 1A. (B) Head CT analysis revealed subdural hematoma (indicated by arrow) during induction therapy. (C) Clinical course of case 3 after mCR that achieved at 4 months after initial diagnosis. The provided treatments are shown in the upper panel. Graphs show PML‐RARA copy number in the BM or CSF, and WBC number in the CSF as indicated. (D) Morphologic analyses of the WBC cells from the CSF by Giemsa staining (1000×).

The patient was 70‐year‐old male at the initial diagnosis. Peripheral WBC count was 16.6 × 10^9^/L, platelet count was 9 × 10^9^/L, and his bone marrow was occupied with abnormal promyelocytes up to 82%. Chromosomal analysis revealed 46 XX, t(15;17)(q22;q21) without additional chromosomal abnormalities. The isoform of the PML‐RARA fusion gene transcript was not tested. Based on these findings, he was diagnosed with APL classified in the high‐risk group by Sanz score 5. The entire clinical course is summarized in Figure 3A. In brief, a prior chemotherapy course (DNR 50 mg/m^2^ for 5 days and cytarabine 100 mg/m^2^ for 7 days) was started for cytoreduction, and ATRA was added from day 3. However, on day 5, he presented with convulsions of limbs and bloody phlegm. A CT scan revealed a subdural hematoma (Fig. 3B) and alveolar bleeding. Therefore, the chemotherapy except for ATRA was discontinued. Consequently, he failed to achieve hematological CR in the first induction therapy, and two additional courses of chemotherapy were required to acquire mCR. Two courses of ATO (0.15 mg/kg/day in 5 days a week for 5 weeks) and twelve courses of Am80 (6 mg/m^2^ orally for 14 days) were administrated to maintain CR, and in addition, TIT (methotrexate 15 mg, cytarabine 40 mg, and prednisolone 10 mg) was administrated five times to prevent CNS involvement of abnormal promyelocytes (Fig. 3C). However, mCR was lost 20 months after the initial diagnosis and, 10 months later, had hematological relapse and simultaneous CNS involvement of abnormal promyelocytes without additional chromosomal abnormalities (Fig. 3C). ATRA and GO failed to reduce abnormal promyelocytes in the BM and CNS, while re‐administration of ATO was still able to eliminate abnormal promyelocytes from the BM, but not from the CNS. Finally, as it was impossible to safely perform TIT due to a technical difficulty in accessing his medullary cavity for an unknown reason, his CNS disease was controlled using CSI (44.8 Gy in total). He is currently alive with mCR in the BM and without neurological symptoms, although his CSF test has not been performed owing to technical difficulties in safely drawing CSF.

## Discussion

Two large cohort studies of APL including GIEMEMA (AIDA 0493) and European‐PETHEMA (APL91, APL93, and PETHEMA96) reported that the frequency of CNS involvement among all relapse cases was 7.6% and 5.3%, respectively 4, 8. Our institution had three (42.9%) CNS involvement cases of seven relapsed cases in the last quarter of a century (Table 1). This high rate of CNS involvement might be due to an institutional bias. Anyhow, the current two cases (case 2 and case 3) with simultaneous occurrence of CNS involvement and first hematological recurrence might suggest that we should take CNS involvement into consideration even in cases with first hematological recurrence.

**Table 1:** APL relapse cases experienced in our institution between 1991 and 2015

Sex/Age (years)	Cytogenesis	WBC (x 10^9^/L)	Risk group (Sanz score)	CD56	DS	CNS prohylaxis	PML‐RARA isoform	CNS bleeding	Time to 1st hematological relapse	Time to CNS invavsion/relapse	1st induction therapy
M/68	46,XY,t(15;17)(q22;q21)	3.4	Intermediate	N	No	No	bcr3	No	16 M	16 M	ATRA+CT
M/50	48,XY,+1,+8,del(11)(p 15)add(11) (q13),t(15;17)(q22;q21)	1.2	Low	N	No	No	NT	No	14 M	–	ATRA
F/22	46,XX,t(15;17)(q22;q21)	1.1	Low	NT	No	No	NT	No	9 M	–	CT
M/63	46,XY,t(15;17)(q22;q21)	0.5	Low	P	No	No	bcr3	No	10 M	–	ATRA
F/49	46XX,t(15;17)(q22;q21)	15.2	High	NT	No	No	bcr3	No	14 M	–	ATRA+CT
F/57	46,XX,t(15;17)(q22;q21)	0.8	Low	N	Yes	No	bcr3	No	13 M	90 M (54 M after allo‐SCT)	ATRA+CT
M/70	46,XY,t(15;17)(q22;q12)	16.6	High	P	No	Yes (TIT)	NT	Yes	30 M	30 M	ATRA+CT

M in sex, male; F in sex, female; WBC, white blood cell count; CNS, central nervous system; APL, acute promyelocytic leukemia; DS, differentiation syndrome; TIT, triple intrathecal therapy; BM, bone marrow; CR, complete remission; ATRA, all‐trans retinoic acid; CT, chemotherapy; allo‐SCT, allogeneic stem cell transplantation; NT, not tested; M in time, month.

Central nervous system involvement in APL has been reported to be associated with several factors including high WBC count (>10 × 10^9^/L) at initial diagnosis, CNS hemorrhage, expression of CD2, and/or CD56 in abnormal promyelocytes, PML‐RARA bcr3 isoform, differentiation syndrome, use of monotherapy‐only regimens, and use of regimens that exclude cytarabine 4, 9, 10, 11, 12, 13, 14. The laboratory characteristic that was shared in more than one case in the current report is the PML‐RARA isoform bcr3 (case 1 and case 2). Previous reports described that the bcr3 isoform is associated with high frequency of relapse and poor clinical outcome 15, 16. In fact, bcr3 was the PML‐RARA isoform in all the four relapse cases that we could investigate. These findings indicate that the bcr3 isoform might be a risk factor for systemic recurrence rather than only for CNS involvement. In addition, it is unclear whether additional genetic events could be indispensable for CNS involvement because additional chromosomal abnormalities were not detected when we noticed CNS involvement. Case 3 presented multiple factors including high WBC count at initial diagnosis, CNS hemorrhage, and expression of CD56 in abnormal promyelocytes. The high WBC count at initial diagnosis and expression of CD56 in abnormal promyelocytes might not be definitive risk factors for CNS involvement because individual patients who had either factor did not develop CNS involvement (Table.1). An obvious infiltration of abnormal promyelocytes by CNS hemorrhage during early phase of disease may be a risk of CNS involvement. It is unclear whether cytarabine‐free treatments as administrated in case 2 could be a risk factor for CNS involvement, because cytarabine had been administrated in case 1 and case 3. Further studies would be required to more robustly identify risk factors for CNS involvement.

The best way to eliminate abnormal promyelocytes from CNS has not been established yet. Case 1 and case 2 demonstrated that a direct administration of anticancer drugs into medullary cavity could successfully induced mCR in the CNS, indicating that TIT is effective and may be recommended as the first treatment for CNS involvement. In contrast, ATO seems to be ineffective for CNS involvement although it has been reported to be able to enter into CNS 17. In case 1, the initial CNS infiltration of abnormal promyelocytes was uncovered during the maintenance of mCR in the BM by repeated ATO administration, suggesting that ATO administration presumably does not prevent CNS invasion of APL cells. In case 2, ATO was effective for hematological recurrence and induced mCR in the BM. However, after cessation of TIT, the PML‐RARA transcript level increased again in the CSF but not in the BM despite continuous administration of ATO, indicating that ATO is insufficient in controlling CNS involvement. GO is a calicheamicin‐conjugated humanized anti‐CD33 monoclonal antibody, which targets CD33 that is highly expressed on the surface of abnormal promyelocytes 18. However, because GO is not able to penetrate into CSF, it is thought to be ineffective for abnormal promyelocytes in CSF as observed in case 2 and case 3. The efficacy of GO observed in case 1 might be because of a transient damage of the blood‐brain barrier due to biopsy examination. Am80 is a synthetic retinoid and exerts an effect comparable with that of ATRA in maintenance therapy. However, it is uncertain whether Am80 could penetrate into CNS. The current report does not provide any information about the efficacy of Am80 for CNS involvement because mCR was lost during Am80 treatment before abnormal promyelocytes were detected in the CNS in case 2. CNS prophylaxis by TIT was administrated in case 3 because this patient possessed multiple risk factors for CNS involvement. However, this patient unfortunately developed CNS involvement 14 months after cessation of TIT. Therefore, it is difficult to determine whether CNS prophylaxis was meaningless. CSI was clearly effective for second CNS relapse after multiple repetitions of TIT as observed in case 2. In cases that the disease is limited to the CNS, CSI might be a potential therapeutic strategy although the possibility of adverse effect, such as leukoencephalopathy, should be carefully considered.

In summary, CSF test might be considerable in APL patients with hematological recurrence. CNS involvement appears to respond well to TIT and CSI but not to ATO. Significance of CNS prophylaxis remains unclear. Further studies would be required to address the best strategy to prevent CNS involvement in APL.

## Authorship

AF: collected clinical data and wrote the manuscript. MK: wrote the manuscript and supervised the study. MK, YO, AU, SN, SH, and HM: treated the patients and collected partial clinical data. KK and AA: supervised the study.

## Conflict of Interest

The authors declare no conflict of interest.
